# Is COVID‐19 Vaccination Beneficial for Tumor Patients: A Cross‐Sectional Investigation in China

**DOI:** 10.1002/iid3.70069

**Published:** 2024-11-27

**Authors:** Sixiu Wang, Yan Zhu, Tao Chen, Chunying Lin, Liming Chen, Yongdong Niu, Congzhu Li

**Affiliations:** ^1^ School of Public Health Shantou University Shantou China; ^2^ Department of Gynecologic Oncology Cancer Hospital of Shantou University Medical College Shantou China; ^3^ Zhongshan Medical School Sun Yat‐sen University Guangzhou China; ^4^ Department of Oncology First Affiliated Hospital of Shantou University Medical College Shantou China; ^5^ Department of Pharmacology, School of Medicine Shantou University Shantou China

**Keywords:** benefits, COVID‐19, tumor, uptake, vaccination

## Abstract

**Introduction:**

Tumor patients take a high risk of SARS‐CoV‐2 infection, high incidence of serious events, poor prognosis and high mortality in the coronavirus disease 2019 (COVID‐19) epidemic, but there is still lack of supporting evidence that the COVID‐19 vaccination is beneficial for tumor patients to encourage them to receive the vaccination.

**Methods:**

A cross‐sectional study was conducted in Shantou, China and questionnaires were collected in the hospitals from February 13, 2023 to April 23, 2023. Using the receiving of COVID‐19 vaccination as the primary outcome, descriptive, univariate and multivariate analyses were generated.

**Results:**

161 out of 241 patients (66.80%) had received at least one dose of COVID‐19 vaccine and 61.00% patients had been infected with SARS‐CoV‐2. Patients with general symptoms (*p* = 0.013) and others (*p* = 0.022) had a higher proportion of nonvaccinated patients than vaccinated ones. In the multivariate analysis, age (aOR = 0.971, 95% CI = 0.946–0.997, *p* = 0.031), the cognition of vaccines' impact on tumor treatment (aOR = 4.475, 95% CI = 1.772–11.299, *p* = 0.002), time since tumor diagnosis (aOR = 4.586, 95% CI = 2.122–9.909, *p* < 0.001) were identified as factors of COVID‐19 vaccination uptake.

**Conclusion:**

COVID‐19 vaccination in China offers numerous advantages for tumor patients, helping to alleviate symptoms following infection and potentially decreasing the chances of tumor metastasis and recurrence.

## Graphical abstract

Tumor patients take a high risk of corona virus 2 (SARS‐CoV‐2) infection, high incidence of serious events, poor prognosis and high mortality in the coronavirus disease 2019 (COVID‐19) epidemic, but there is still lack of more vaccination beneficial supporting evidence to encourage tumor patients to receive COVID‐19 vaccination. We hope that our findings will strengthen their confidence of vaccines and improve the vaccination rate of them, so as to fill the gap in the immunization level for tumor patients and further reduce the risk of severe illness and death.

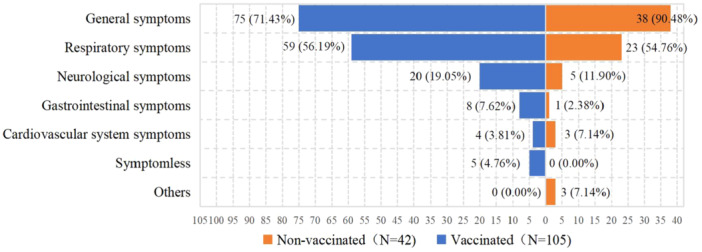

## 1 Introduction

Severe acute respiratory syndrome corona virus 2 (SARS‐CoV‐2) has caused the epidemic of coronavirus disease 2019 (COVID‐19), and vaccination is an important way to prevent COVID‐19. Chinese government launched COVID‐19 vaccination on December 15, 2020, which is free for all [1]. As of January 30, 2023, more than 90 percent of the population had been fully vaccinated in China [2]. On December 7, 2022 China's National Health Commission (NHC) released a circular on further optimizing its COVID‐19 response, announcing 10 new prevention and control measures to ease restrictions on visits to public venues and travel [3]. Subsequently, the Chinese Center for Disease Control and Prevention reported that the positive rate of nucleic acid testing (29.2%) peaked on December 25 and that of antigen testing (21.3%) peaked on December 22.2.

At present, the focus of vaccination is to fill the gap in the immunization level for different target populations and further reduce the risk of severe illness and death. The populations include individuals with high risk of infection, the elderly over 60 years old, people with serious underlying diseases and those with low immunity [4]. Tumor patients were usually in a high risk of infection exposure, with high incidence of serious events, poor prognosis and high mortality in the COVID‐19 epidemic [5, 6]. However, there is still lack of the positive feedback on the doubts and worries about their common and/or advancing cancer therapy and their adverse reactions after COVID‐19 vaccination [7]. Therefore, their acceptance rate of COVID‐19 vaccination is low [8]. According to Chinese Ministry of Foreign Affairs, Chinese vaccines have been supplied to more than 100 countries and international organizations [9]. In reality, studies have reported the safety and immunogenicity for tumor patients to receive COVID‐19 inactivated vaccines [10, 11, 12]. COVID‐19 vaccines in China are mainly produced and developed by Sinopharm [13], Sinovac [14, 15], Consino [16, 17], Chongqing Zhifei [18]. and Shenzhen Kangtai [19], of which most are inactivated vaccines and almost none is messenger RNA (mRNA) vaccine, while longevity of immunity in preventing severe infection outcomes has been observed in tumor patients received COVID‐19 mRNA vaccines and mRNA vaccine is now as a powerful tool for treating malignant tumors thanks to the remarkable clinical outcomes of mRNA vaccines during the COVID‐19 pandemic [19, 20]. Correspondingly, could inactivated vaccine stimulate specific cellular immunity and humoral immune responses to prevent tumor growth? It is still not clear.

In this study, an attempt was made to investigate the attitudes of tumor patients towards COVID‐19 and their status of COVID‐19 vaccination after the peak period of SARS‐CoV‐2 infection in China in December 2022. The purpose is to find out the doubts of tumor patients about COVID‐19 vaccine and to further explore the factors or benefits of vaccination among tumor patients, so as to strengthen their confidence of vaccines and propose targeted methods to improve the vaccination rate of them.

## 2 Methodology

### 2.1 Study Design

We identified tumor patients from the Oncology Department of First Affiliated Hospital of Shantou University Medical College and the Gynecology Department of Cancer Hospital of Shantou University Medical College for the cross‐sectional study. Based on the literature and expert opinions, we designed our own questionnaire. A pilot study had been conducted before the formal initiation of the study. The content of questionnaire was refined based on feedback from 36 participants, which has four parts. The first three parts include the basic information of the patient, the related cognition and situation of COVID‐19 and the cognition of the disease, and in the fourth part, the disease situation is provided by the medical staff. Finally, we conducted a paper version of the questionnaire in the outpatient and inpatient departments of the hospital from February 13, 2023 to April 23, 2023, all of which were filled out by the investigators, patients or their families under the introduction and explanation of the investigators. No incentives were provided. Inclusion criteria to recruit participants were people aged ≥ 18 years; people had a confirmed tumor; people had case records in hospital; and those who voluntarily agreed to participate in the present study. Exclusion criteria were who could not understand the content of the questionnaire after the investigator explained.

### 2.2 Sample Size Estimation

This is an exploratory study, and we used a cross‐sectional design to estimate the sample size.

$$n=\frac{Z_{1-\alpha /2\;}^{2}\times \;pq}{d^{2}}.$$

The COVID‐19 vaccination rate of the 36 participants in the Pilot survey was 50.00%. In the formula, *p* = 1‐*q*. Assuming significance *a* = 0.05 (bilateral), limit error *d* = 0.15*p*, where *p* is the vaccination rate, so the sample size should be 171.

Taking into account the invalid questionnaire and extending the calculated sample size by 20%, the total sample size required is 206.

### 2.3 Variables

The study obtained the information we wanted through the questionnaire survey, the hospital medical record system and the COVID‐19 vaccination information platform “Yueshengshi” (managed by the Guangdong provincial government). The content of the questionnaire was modified and determined by three experts in the field of oncology to ensure the professionalism of the questionnaire content.

In the questionnaire survey, we collected the basic information variables, including age, education level, work status, marital status, monthly income, insurance status, smoking status and drinking status.

To assess the attitudes of tumor patients towards COVID‐19, we set the following questions in the questionnaire: (1) “Do you know anything about COVID‐19?” (2) “Do you worry about infecting with COVID 19?” (3) “Has COVID‐19 pandemic had an impact on your family's economy?” For the first two questions, we set the answer to the Likert scale from 1 to 5 representing respectively very strong, much, rather, a little, not at all.

Regarding the variables about tumor patients receiving the COVID‐19 vaccine, we ask the participants whether they had been vaccinated and the number of doses. In addition, to ensure accuracy of the self‐reported information, including the type and time of the vaccination, the related information from participants who had been vaccinated were asked to check their vaccine records also through their “Yueshengshi” APP, which had been used to collect health information and inconsistent recognition of isolation between regions by pooling information including COVID‐19 screening results, temperature check records, health declaration records, etc. In addition, the questionnaire included whether patients have adverse reactions after vaccination, whether they are willing to receive or continue receiving vaccination, and reasons for the different willingness. Those who have no willing to vaccinate, were asked that if the attending physician recommends them to vaccinate, whether they will change their mend.

The history of SARS‐CoV‐2 infection was also asked, including whether participants infected, whether their families infected and whether purchased COVID‐19 specific medicine. In addition, the infected ones filled in the test methods, the time of the infection, symptoms (multiple choice), whether they took the drug and saw a doctor. The symptoms including symptomless, general symptoms (fever, muscule soreness, fatigue, tired), cardiovascular system symptoms (chest tightness, chest pain, palpitation), respiratory symptoms (rhinobyon, rhinorrhea, cough, expectoration, chest radiograph or CT showed pulmonary inflammation, dysplessness or shortness of breath), neurological symptoms (memory and attention problems, Insomnia, dizziness, headache, depression or anxiety, hypograde or loss of olfactory taste, conjunctivitis, xeroma, sudden deafness, diminution of vision), gastrointestinal symptoms (loss of appetite, nausea and vomiting, dysphagia, abdominal pain and diarrhea) and others (responses outside of the options).

Finally, we learned the patient's condition from themselves and their doctors respectively. From the patient's point of view, their health status before the diagnosis of the tumor were self‐evaluated by using Likert scale. Other chronic diseases, symptoms of discomfort during tumor, the condition after treatment, the possibility of a tumor cure, the current charges for treatments, and the willingness to try other methods that may help with your disease were also asked. From the doctors' perspective, we gained the patients' conditions through the hospital medical record system, including patient's tumor name, time of the first tumor diagnosis, tumor stage (Tumor Node Metastasis [TNM]/International Federation of Gynecology and Obstetrics), treatment methods, the date of treatment methods, metastasis, and recurrence.

### 2.4 Statistical Methods

After entering data with EpiData version 3.1, random forest multiple imputation, implemented using the MissForest 20 package in R version 4.3.0, was used to impute values for missing values. Using the receiving of COVID‐19 vaccination as the primary outcome, descriptive, univariate and multivariable analyses were generated in this study and conducted by IBM Statistical Package for the Social Sciences (SPSS) version 23.0. Descriptive results were expressed as percentages for categorical variables and as mean values ± SD or medians [IQR] for continuous variables, as appropriate. The Chi‐square test or Fisher's exact test was applied to compare the unordered categorical variables and ordinal categorical variables were compared using Kruskal‐Wallis test. Student's *t*‐test or nonparametric test was applied to compare the continuous variables, as appropriate. Multivariate logistic regression analyses were used to evaluate the predictors for receiving of COVID‐19 vaccination (1 is assigned to those who were vaccinated and 0 is assigned to those who were unvaccinated) and included variables with *p* < 0.05 in the univariate analysis.

## 3 Results

### 3.1 Demographic Characteristics of the Samples

In total, 241 eligible patients completed the questionnaire in this survey. The summary of participants' demographic characteristics is provided in Table 1. Of the 241 participants included, the age ranged from 28 to 85 years (median: 59 years, interquartile range: 50.00, 68.00). There were 72 male patients (29.88%) and 169 female patients (70.12%). The respondents mainly with a primary school or below (56.02%), were married (88.80%), had an average monthly income of CNY less than ¥1000 (52.28%), had medical insurance (95.85%), had no smoking (79.25%) and had no drinking (90.46%). Only 22.82% had jobs, and the others were unemployed (34.44%) or retired (42.74%). 161 (66.80%) patients, who reported that they had already received COVID‐19 vaccine, were significantly younger than those who had not received (*p* = 0.02).

**Table 1:** Basic characteristics of tumor patients.

Variable	Total (*N* = 241), *n* (col%)	Vaccinated (*N* = 161), *n* (col%)	Nonvaccinated (*N* = 80), *n* (col%)	*p* value
**Age, medians** (IQR)	59.00 (50.00, 68.00)	57.00 (49.50, 66.50)	62.00 (52.00, 69.75)	0.020
**Gender**				0.221
Male	72 (29.88)	44 (27.33)	28 (35.00)	
Female	169 (70.12)	117 (72.67)	52 (65.00)	
**Education level**				0.753
Primary school or below	135 (56.02)	93 (57.76)	42 (52.50)	
Junior high school	47 (19.50)	31 (19.25)	16 (20.00)	
Senior high school	37 (15.35)	22 (13.66)	15 (18.75)	
College degree or above	22 (9.13)	15 (9.32)	7 (8.75)	
**Work status**				0.467
Employed	55 (22.82)	40 (24.84)	15 (18.75)	
Unemployed	83 (34.44)	56 (34.78)	27 (33.75)	
Retired	103 (42.7)	65 (40.37)	38 (47.50)	
**Marital status**				0.395
Unmarried/Divorced/Widowed	27 (11.20)	20 (12.42)	7 (8.75)	
Married	214 (88.80)	141 (87.58)	73 (91.25)	
**Monthly income (CNY)**				0.321
< ¥1000	126 (52.28)	84 (52.17)	42 (52.50)	
¥1000–4999	94 (39.00)	60 (37.27)	34 (42.50)	
≥ ¥5000	21 (8.71)	17 (10.56)	4 (5.00)	
**Insurance status**				1.000
No	10 (4.15)	7 (4.35)	3 (3.75)	
Yes	231 (95.85)	154 (95.65)	77 (96.25)	
**Smoking status**				0.777
Yes	26 (10.79)	16 (9.94)	10 (12.50)	
No	191 (79.25)	128 (79.50)	63 (78.75)	
Ex‐smoker	24 (9.96)	17 (10.56)	7 (8.75)	
**Drinking status**				0.767
Yes	8 (3.32)	6 (3.73)	2 (2.50)	
No	218 (90.46)	146 (90.68)	72 (90.00)	
Ex‐drinker	15 (6.22)	9 (5.59)	6 (7.50)	

Abbreviations: CNY, Chinese Yuan; IQR, interquartile range.

### 3.2 Vaccination Status

**Figure 1:**
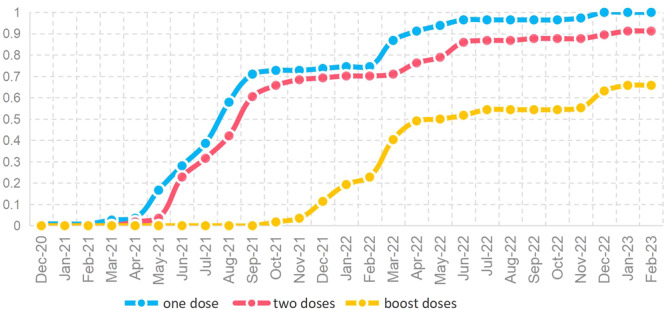
The trend of different doses of vaccination rates among tumor patients who had vaccinated. The cumulative vaccination rate for one dose apparently rose from April to September 2021 and from February to May 2022; The cumulative vaccination rate for two doses followed that for one; The cumulative vaccination rate for boost doses apparently rose from November 2021 to April 2022 and in October 2022.

As of the time of the survey, 161 (66.80%) have been vaccinated and 80 (33.20%) have not. A total of 111 (40.06%) received booster injections (three or four dose), 39 (16.18%) received two doses, and 11 (4.56%) received only one dose. 23 (14.28%) reported adverse events following immunization (AEFI), including thirteen fatigue, eleven muscule soreness, three headache, two fever, two rash, one sore throat, one nausea and vomiting, one thirsty, one nosebleed, one chest pain, one heart discomfort and one diarrhea. The complete vaccination information was provided by 114 patients, accounting for 70.81% of the vaccinated population, where 106 (92.98%) received only inactivated vaccine, two (1.75%) received only recombinant protein vaccine, one (0.88%) received only adenovirus vector vaccine, and five (4.39%) received heterologous vaccine. The trend of different dose of vaccination rates among 114 patients showed in Figure 1.

### 3.3 SARS‐CoV‐2 Infection

**Figure 2:**
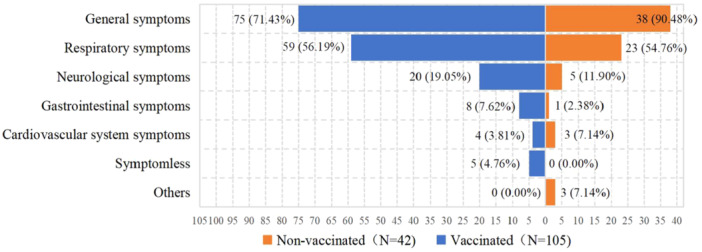
The symptoms after SARS‐CoV‐2 infection.

Based upon participants' memories, 147 (61.00%) had been infected with SARS‐CoV‐2, and 94 (39.00%) were not. 90 (37.34%) had nucleic acid or antibody test positive. 57 (23.65%) said they had suspected symptoms and speculated that they had been infected. There was no statistical difference (*p* = 0.057) in infection rates between the vaccinated (65.22%) and non‐vaccinated (52.50%) groups. The time of their infection ranged from November 2022 to February 2023, which was concentrated in December 2022 (73.47%) and January 2023 (19.73%). The infections among the members of vaccinated group all occurred after vaccination. The Symptoms after the infection were general symptoms (76.87%), respiratory symptoms (55.78%), neurological symptoms (17.01%), gastrointestinal symptoms (6.12%), cardiovascular system symptoms (4.76%), symptomless (3.40%) and others (2.04%). Figure 2 illustrates the postinfection symptoms of the different vaccination groups. Patients with general symptoms (*p* = 0.013) and others (*p* = 0.022) had a higher proportion of non‐vaccinated patients than vaccinated ones. 90 (61.22%) patients had taken medicine and 12 (0.08%) patients saw a doctor due to the infection, none related to vaccination. Among all participants, the families of 178 (73.86%) patients were infected with SARS‐CoV‐2 and no one had bought COVID‐19 specific medicine.

### 3.4 Willingness to Receive COVID‐19 Vaccine

Only 44 (18.26%) participants were willing to receive or continue receiving vaccination and the top two reasons (multiple selections) were “COVID‐19 vaccine is safe and effective” (68.18%), “COVID‐19 vaccine is free” (27.27%). Self‐reported reasons (multiple selections) for 197 (81.74%) patients unwilling to get vaccinated were “Fear of affecting their disease” (31.98%), “Concerns about AEFI” (29.95%), “Fear of affecting their treatments” (24.37%), “Have contraindications” (14.72%), “No strong recommendation from doctors” (13.20%), and “others” (19.29%). The proportion of willingness was 5.00% in unvaccinated group, 23.08% in one dose group, 16.28% in two dose group and 28.57% in boost doses group (*p* = 0.001). Furthermore, if the attending physician suggests to receive vaccination, 78 (39.59%) previously reluctant participants would be willing to be vaccinated.

### 3.5 Cognition and Attitude Toward COVID‐19 and COVID‐19 Vaccines

When involving cognition and attitude towards COVID‐19, 111 (46.06%) participants' knowledge of COVID‐19 was 2 (much), 94 (39.00%) and 86 (35.68%) participants' degree of concern about infecting with COVID 19 was 4 (a little) and 3 (rather), and 111 (46.06%) reported COVID‐19 pandemic had an impact on their family's economy. When involving cognition and attitude towards COVID‐19 vaccines, 139 (57.68%) affirmed COVID‐19 vaccines can be useful in controlling the COVID‐19 pan‐demic. 130 (53.94%) and 139 (57.68%) responded respectively that the vaccine is safe and effective. 37 (15.35%) patients indicated COVID‐19 vaccine could affect regular medical treatment of tumor. For this point, 65 (40.37%) in the vaccinated group and 15 (18.75%) in the non‐vaccinated group denied respectively (*p* < 0.01). 95 (39.42%) participants agreed that the COVID‐19 vaccine may regulate autoimmunity and thus enhance their immunity, while 127 (52.70%) were not sure (Table 2).

**Table 2:** Cognition and attitude toward COVID‐19 and COVID‐19 vaccines.

Variable	Total (*N* = 241), *n* (col%)	Vaccinated (*N* = 161), *n* (col%)	Nonvaccinated (*N* = 80), *n* (col%)	*p* value
**Do you know anything about COVID‐19?**				0.097
1 (Very strong)	26 (10.79)	20 (12.42)	6 (7.50)	
2 (Much)	111 (46.06)	76 (47.20)	35 (43.75)	
3 (Rather)	56 (23.24)	37 (22.98)	19 (23.75)	
4 (A little)	39 (16.18)	24 (14.91)	15 (18.75)	
5 (Not at all)	9 (3.73)	4 (2.48)	5 (6.25)	
**Do you worry about infecting with COVID 19?**				0.752
1 (Very strong)	17 (7.05)	11 (6.83)	6 (7.50)	
2 (Much)	24 (9.96)	17 (10.56)	7 (8.75)	
3 (Rather)	86 (35.68)	54 (33.54)	32 (40.00)	
4 (A little)	94 (39.00)	67 (41.61)	27 (33.75)	
5 (Not at all)	20 (8.30)	12 (7.45)	8 (10.00)	
**Has COVID‐19 pandemic had an impact on your family's economy?**				0.752
Yes	111 (46.06)	73 (45.34)	38 (47.50)	
No	130 (53.94)	88 (54.66)	42 (52.50)	
**Can COVID‐19 vaccine be useful in controlling the COVID‐19 pandemic?**				0.127
Yes	139 (57.68)	100 (62.11)	39 (48.75)	
No	17 (7.05)	11 (6.83)	6 (7.50)	
Not sure	85 (35.27)	50 (31.06)	35 (43.75)	
**Is the COVID‐19 vaccine safe?**				0.136
Yes	130 (53.94)	94 (58.39)	36 (45.00)	
No	22 (9.13)	14 (8.70)	8 (10.00)	
Not sure	89 (36.93)	53 (32.92)	36 (45.00)	
**Is the COVID‐19 vaccine effective?**				0.142
Yes	139 (57.68)	100 (62.11)	39 (48.75)	
No	20 (8.30)	12 (7.45)	8 (10.00)	
Not sure	82 (34.02)	49 (30.43)	33 (41.25)	
**Could COVID‐19 vaccine affect regular medical treatment of tumor?**				0.001
Yes	37 (15.35)	18 (11.18)	19 (23.75)	
No	80 (33.20)	65 (40.37)	15 (18.75)	
Not sure	124 (51.45)	78 (48.45)	46 (57.50)	
**Do you agree that the COVID‐19 vaccine may regulate autoimmunity and thus enhance your immunity?**				0.764
Yes	95 (39.42)	66 (40.99)	29 (36.25)	
No	19 (7.88)	12 (7.45)	7 (8.75)	
Not sure	127 (52.70)	83 (51.55)	44 (55.00)	

### 3.6 Disease Condition

In the questionnaires, 96 (39.83%) tumor patients reported that they had other chronic diseases and 126 (52.28%) patients reported they had symptoms of discomfort during tumor. Most of the patients were better after receiving treatment (47.72%), believed they could get a tumor cure (50.21%), could afford the charges for treatments that help with their tumor (49.79%), and were willing to try other methods that may help with their tumor (80.50%). 76 (31.54%) participants' health status before the diagnosis of the tumor was 3 (rather). According to the hospital medical record system, the majority of patients had a diagnosis of lung cancer (18.67%), followed by cervical cancer (15.35%), ovarian cancer (11.62%) and breast cancer (11.20%), and most of them were diagnosed in 2022 (28.63%) and 2023 (20.33%). In terms of treatment, the proportion of these who had received operation, chemotherapy, radiotherapy, targeted therapy, immunotherapy and endocrine therapy was 62.24%, 53.94%, 15.77%, 30.29%, 14.52% and 7.47%, respectively. 35 (14.52%) patients had experienced tumor metastasis or recurrence after their treatments. The vaccination status was significantly associated with the possibility of a tumor cure, time since tumor diagnosis (year), tumor stage, targeted therapy and metastasis or recurrence (Table 3).

**Table 3:** Disease condition.

Variable	Total (*N* = 241), *n* (col%)	Vaccinated (*N* = 161), *n* (col%)	Nonvaccinated (*N* = 80), *n* (col%)	*p* value
**Other chronic diseases**				0.551
No	145 (60.17)	99 (61.49)	46 (57.50)	
Yes	96 (39.83)	62 (38.51)	34 (42.50)	
**Symptoms of discomfort during tumor**				0.617
No	115 (47.72)	75 (46.58)	40 (50.00)	
Yes	126 (52.28)	86 (53.42)	40 (50.00)	
**The condition after receiving treatment**				0.435
Better	115 (47.72)	81 (50.31)	34 (42.50)	
Stable	80 (33.20)	48 (29.81)	32 (40.00)	
Worse	8 (3.32)	5 (3.11)	3 (3.75)	
Not sure	38 (15.77)	27 (16.77)	11 (13.75)	
**Health status before the diagnosis of the tumor**				0.196
1 (Very strong)	33 (13.69)	22 (13.66)	11 (13.75)	
2 (Much)	58 (24.07)	41 (25.47)	17 (21.25)	
3 (Rather)	76 (31.54)	55 (34.16)	21 (26.25)	
4 (A little)	65 (26.97)	38 (23.60)	27 (33.75)	
5 (Not at all)	9 (3.73)	5 (3.11)	4 (5.00)	
**The possibility of a tumor cure**				0.027
Can be cured	121 (50.21)	88 (54.66)	33 (41.25)	
Can maintain the status quo	64 (26.56)	33 (20.50)	31 (38.75)	
Can not be cured	17 (7.05)	12 (7.45)	5 (6.25)	
Not sure	39 (16.18)	28 (17.39)	11 (13.75)	
**How do you feel about the current charges for treatments that help with your disease?**				0.050
Can afford	120 (49.79)	88 (54.66)	32 (40.00)	
Can afford the part	89 (36.93)	51 (31.68)	38 (47.50)	
Can not afford	32 (13.28)	22 (13.66)	10 (12.50)	
**Are you willing to try other methods that may help with your disease?**				0.629
Yes	194 (80.50)	131 (81.37)	63 (78.75)	
No	47 (19.50)	30 (18.63)	17 (21.25)	
**Tumor type**				0.065
Benign/borderline tumor	17 (7.05)	14 (8.70)	3 (3.75)	
Thoracic cancer	81 (33.61)	47 (29.19)	34 (42.50)	
Abdominal cancer	50 (20.75)	33 (20.50)	17 (21.25)	
Gynecologic cancer	81 (33.61)	61 (37.89)	20 (25.00)	
Other type of cancer	12 (4.98)	6 (3.73)	6 (7.50)	
**Time since tumor diagnosis (year)**				< 0.001
2019 or before	54 (22.4)	31 (19.3)	23 (28.8)	
2020‐2021	66 (27.4)	28 (17.4)	38 (47.5)	
2022‐2023	121 (50.2)	102 (63.4)	19 (23.8)	
**Tumor stage**				0.021
Benign～Ⅰ	48 (19.92)	38 (23.60)	10 (12.50)	
Ⅱ	26 (10.79)	21 (13.04)	5 (6.25)	
Ⅲ	46 (19.09)	33 (20.50)	13 (16.25)	
Ⅳ	81 (33.61)	45 (27.95)	36 (45.00)	
Unknown	40 (16.60)	24 (14.91)	16 (20.00)	
**Operation**				0.613
Yes	150 (62.24)	102 (63.35)	48 (60.00)	
No	91 (37.76)	59 (36.65)	32 (40.00)	
**Chemotherapy**				0.060
Yes	130 (53.94)	80 (49.69)	50 (62.50)	
No	111 (46.06)	81 (50.31)	30 (37.50)	
**Radiotherapy**				0.545
Yes	38 (15.77)	27 (16.77)	11 (13.75)	
No	203 (84.23)	134 (83.23)	69 (86.25)	
**Targeted therapy**				0.021
Yes	73 (30.29)	41 (25.47)	32 (40.00)	
No	168 (69.71)	120 (74.53)	48 (60.00)	
**Immunotherapy**				0.189
Yes	35 (14.52)	20 (12.42)	15 (18.75)	
No	206 (85.48)	141 (87.58)	65 (81.25)	
**Endocrine therapy**				0.116
Yes	18 (7.47)	9 (5.59)	9 (11.25)	
No	223 (92.53)	152 (94.41)	71 (88.75)	
**Metastasis or recurrence**				0.004
Yes	35 (14.52)	16 (9.94)	19 (23.75)	
No	206 (85.48)	145 (90.06)	61 (76.25)	

### 3.7 Factors Associated With Vaccination and NonVaccination

In the multivariate logistic regression analysis (Table 4), the value of *p* for the Hosmer and Lemeshow test was 0.119, suggesting an acceptable fit. Seven factors associated with COVID‐19 vaccination among tumor patients were included in that analysis. Age, measured as the continuous variable, was identified as a significant impact factor, and older respondents were less likely to have received COVID‐19 vaccines (adjusted odds ratio [aOR] = 0.971, 95% CI = 0.946–0.997, *p* = 0.031). The respondents who disagreed the point that COVID‐19 vaccine could affect regular medical treatment of tumor were more likely to have received vaccines than those who agreed that point (aOR = 4.475, 95% CI = 1.772–11.299, *p* = 0.002). Patients diagnosed with tumor in 2022 and 2023 were more likely to have received vaccines than those diagnosed in 2019 or before (aOR = 4.586, 95% CI = 2.122–9.909, *p* < 0.001).

**Table 4:** Univariate and multivariate logistic regression of characteristics for association with vaccination and nonvaccination.

Variable	Univariate logistic regression analysis		Multivariate logistic regression analysis	
	Crude odds ratio (95% CI)	*p* value	Adjusted odds ratio (95% CI)	*p* value
**Age**	0.975 (0.953–0.998)	0.032	0.971 (0.946–0.997)	0.031
**Could COVID‐19 vaccine affect regular medical treatment of tumor?**				
Yes	Reference		Reference	
No	4.574 (1.946–10.754)	< 0.001	4.475 (1.772–11.299)	0.002
Not sure	1.790 (0.854–3.753)	0.121	1.932 (0.847–4.406)	0.118
**The possibility of a tumor cure**				
Can be cured	Reference		NA	NA
Can maintain the status quo	0.399 (0.212–0.752)	0.004	NA	NA
Can not be cured	0.900 (0.294–2.751)	0.853	NA	NA
Not sure	0.955 (0.427–2.133)	0.910	NA	NA
**Time since tumor diagnosis (year)**				
2019 or before	Reference		Reference	
2020–2021	0.547 (0.264–1.132)	0.104	0.618 (0.283–1.346)	0.226
2022–2023	3.983 (1.922–8.253)	< 0.001	4.586 (2.122–9.909)	< 0.001
**Tumor stage**				
Benign～Ⅰ	Reference		NA	NA
Ⅱ	1.105 (0.333–3.664)	0.870	NA	NA
Ⅲ	0.668 (0.163–1.722)	0.404	NA	NA
Ⅳ	0.329 (0.144–0.749)	0.008	NA	NA
Unknown	0.395 (0.154–1.011)	0.053	NA	NA
**Targeted therapy**				
No	Reference		NA	NA
Yes	1.951 (1.103–3.453)	0.022	NA	NA
**Metastasis or recurrence**				
No	Reference		NA	NA
Yes	2.823 (1.361–5.853)	0.005	NA	NA

Abbreviation: NA, no statistical significance.

### 3.8 The Possible Benefits of Vaccination

Factors including metastasis or recurrence, symptoms after the infection can reflect whether the inactivated vaccines have a positive impact on tumor patients. Metastasis or recurrence (*p* = 0.004) and part of the symptoms (general symptoms, *p* = 0.013; others, *p* = 0.022) after the infection in tumor patients showed statistically significant differences in the vaccinated and non‐vaccinated groups. The proportion of tumor metastasis or recurrence after tumor diagnosis in the vaccinated and non‐vaccinated groups was 9.94% and 23.75%, respectively. Of the 35 patients with metastasis or recurrence, 8 were vaccinated before that, 6 vaccinated after that, 2 had unknown timing, and 19 were non‐vaccinated. Patients with general symptoms (fever, muscule soreness, fatigue, tired) and others (responses outside of the options) had a higher proportion of non‐vaccinated patients than vaccinated ones. All of three patients who selected others in the symptoms option, were non‐vaccinated and the symptoms were elevated blood sugar, stroke, and lower body weakness. All asymptomatic patients were vaccinated.

## 4 Discussion

To the best of our knowledge, this investigation represents one of the few studies in China providing insight into COVID‐19 vaccination and SARS‐CoV‐2 infection among tumor patients. It was found that the vaccination rate was 66.80% from February 13, to April 23, 2023, which was much lower than that in general Chinese population (more than 90%). 2 Compared to the other vaccination rates of tumor patients, it was higher than that in four Chinese cities (49.9%) [21]. and Xian, China (58.15%) [22], which may be due to temporal differences. However, it was lower than that in Australian (79.7%) [23], Mexico (95%) [24]. Therefore, there was still a lot of room for improvement in the vaccination rate. Judging from the dose of vaccination, 62.24% patients received at least two doses of COVID‐19 vaccination and 40.06% received booster injections (three or four dose), both of which were higher than that in four Chinese cities in different geographic regions between May and June 2022 (58.8% and 29.3%, respectively) [25].

This study has some practical implications for Chinese government to fill the gap in the immunization level for tumor patients and further reduce the risk of severe illness and death.4 In our study, the prevalence of willingness to receive or continue receiving vaccination was only 18.26% and unvaccinated populations had the lowest willingness rate (5.00%), while these proportions were higher in another survey among tumor patients [21]. Also, these tumor patients enlisting in this project had a lower intention to vaccinate than the general population [26, 27]. The decline in willingness to vaccinate may be related to the current infection situation, where 61.00% patients had been infected with SARS‐CoV‐2, and the National Health Commission recommends people who had been infected to receive vaccination after 6 months [28]. Due the complexity and multiplicity of vaccine hesitancy, we found that the conceptions that the COVID‐19 epidemic was over and that SARS‐CoV‐2 was no longer dangerous were popular, thus the acceptability and intentions to take the injection of COVID‐19 vaccines are slowly fading away during the whole investigation. Our results showed that the primary willing to be vaccinated was the safety and effectiveness of COVID‐19 vaccination, and the unwilling was the impacts of the vaccine on their disease and health. Meanwhile, ones disagreed the point that COVID‐19 vaccine could affect regular medical treatment of tumors were more likely to have received vaccines. These results all indicated that tumor patients remain skeptical about the vaccine. In line with previous studies [22], results from our study also showed that inactivated vaccines led the Chinese COVID‐19 vaccine market in tumor patients. Further, 14.28% patients reported AEFI, main of which were fatigue and muscle soreness. In reality, a number of studies have reported the safety and immunogenicity of tumor patients to receive inactivated vaccine against SARS‐CoV‐2 10‐12 and booster vaccination provided a high level of protection against pneumonia with SARS‐CoV‐2 infection [25].

In our study, all asymptomatic patients were vaccinated. Patients with general symptoms (fever, muscle soreness, fatigue, tired) and others (responses outside of the options) had a higher proportion of non‐vaccinated than vaccinated patients after SARS‐CoV‐2 infection. All of three patients who selected others in the symptoms option, were non‐vaccinated and the symptoms were elevated blood sugar, stroke, and lower body weakness. In a review in Europe, compared with unvaccinated controls, boosted and vaccinated tumor patients had significant improvements in 14‐day case‐fatality rate (*p* = 0.0011) and 28‐day case‐fatality rate (*p* = 0.015), hospitalization due to COVID‐19 (*p* = 0.0011), and complications from COVID‐19 (*p* = 0.015) [29]. Therefore, we believe that vaccination could reduce the symptoms after infection among tumor patients. In addition, antibody‐dependent enhancement (ADE) in the context of COVID‐19 vaccines has been a matter of debate [30], while our study show that prior immunization did not increase the likelihood of severe diseases. Some serum samples from vaccinated individuals displayed a mild IgA‐ADE effect with Omicron after the second dose of the vaccine, but this effect was abolished after the completion of the full vaccination scheme [31]. Also, the incidence rate ratios for the 3‐dose and 4‐dose group for COVID‐19 hospitalization and severe diseases were proved significantly lower than the 2‐dose group in tumor population [32], so the importance of completing vaccination procedure and booster doses should be emphasized. Accordingly, interventions should be developed to convince tumor patients that the vaccine is safe and effective, and to make tumor patients realize that vaccination, especially the booster doses, can reduce the effects of SARS‐CoV‐2 infection.

Among the influencing factors affecting the acceptance of COVID‐19 vaccine among tumor patients, multivariate analysis showed that age, the cognition of vaccines' impact on tumor treatment and time since tumor diagnosis (year) were different in vaccinated and nonvaccinated group. We found older patients were less likely to have received COVID‐19 vaccines. Previous studies showed that the older tumor patients had lower COVID‐19 vaccination uptake or acceptance [25, 33, 34], but there is no difference in age for vaccination uptake among tumor patients in Guangzhou China [35]. Our results showed that patients who believe COVID‐19 vaccine would not affect their being treatments were 4.475 times more likely to have received vaccines than who didn't think so, which provided more detailed evidence to support earlier research findings of the tumor patient's hesitancy to receive COVID‐19 vaccination. Our results also showed that patients diagnosed with tumor in 2022 and 2023 were 4.586 times more likely to have received vaccines than those diagnosed in 2019 or before. The reasons accounting for the result could be that the new tumor patients had been vaccinated before their diagnosis. In addition, the attitude to the possibility of a tumor cure, tumor stage, targeted therapy and metastasis or recurrence were found to be independently related to vaccination status. Similar to the results that positive attitudes and treatments were contributors to vaccine hesitancy [21, 33], we found that patients who thought their tumors could be effective therapied were more likely to be vaccinated than those who thought they could maintain the status quo, and patients who were in the benign or early stage, had received targeted therapy were also with higher COVID‐19 vaccination rates. Besides, more non‐vaccinated patients developed tumor metastasis or recurrence, where only 10 of the [35]. (Two have no clear time) patients who developed metastasis or recurrence after treatment had been vaccinated before the metastasis or recurrence, which indicated COVID‐19 vaccination could have a positive impact on tumor patients. In another study, 0.69% patients experienced metastasis and recurrence after vaccination, but a retrospective analysis of their disease history showed that they were in the late disease stage at initial diagnosis, with a heavy tumor burden and a poor response to treatment [22]. Our result is similar to that one and metastasis or recurrence in these patients may be due to the tumor rather than vaccination. However, that still indicated COVID‐19 vaccination may have a positive impact on metastasis or recurrence, further studies need to be conducted to draw a solid conclusion. Studies have shown that a woman diagnosed with myoepithelial carcinoma of the left parotid [36]. and a male patient diagnosed with recurrent primary cutaneous anaplastic large‐cell lymphoma [37]. experienced spontaneous tumor regression after COVID‐19 vaccination.

To advocate for vaccination among tumor patients, we recommend alleviating concerns among tumor patients about vaccination affecting treatment and enhance physician advocacy for vaccination. If the attending physician suggests to receive vaccination, 39.59% previously reluctant ones in our study said they were willing to be vaccinated, so physician should incorporate the specific details of each patient and expert advice into an individualized vaccination suggest for tumor patients and encourage them to be vaccinated. When the government and relevant departments publicize the vaccine, to show tumor population more examples that tumor patients gain benefits of vaccination. Further, it can lay a foundation of patients' confidence for the application of vaccines in tumor onco‐immunotherapy.

## 5 Limitations

Several limitations need to be acknowledged. First, our study has a small sample size, and the data were collected in the hospitals in Shantou, China. Therefore, the sample may not represent the geographic, cultural, and socioeconomic variations among Chinese tumor patients. Second, this is a cross‐sectional study, which means that we cannot determine the causality of patients to receive COVID‐19 vaccinations and have a certain degree of selection and recall bias. Third, the benefits of vaccination need to be further validated by survival data.

## 6 Conclusion

After the peak period of the first wave of SARS‐CoV‐2 infection in China, COVID‐19 vaccination brings many benefits to tumor patients, which can reduce symptoms after infection and may even reduce tumor metastasis and recurrence. The vaccine is safe for them and those who were older, thought the vaccines have an impact on tumor treatment and had a longer diagnosis, had lower vaccination rates. In addition, due to the low willingness to receive or continue receiving vaccination, more vaccination education from physicians, more vaccine publicity and onco‐immunotherapy measures are expected to reduce or delay the risk of severe illness and death for tumor patients.

## Author Contributions

Sixiu Wang performed data analysis and drafted the manuscript. Sixiu Wang, Yan Zhu, Liming Chen, Tao Chen, Congzhu Li were all responsible for participant recruitment and data collection. Yongdong Niu, Liming Chen, Yan Zhu and Sixiu Wang designed and edited the study. Liming Chen, Congzhu Li and Yongdong Niu review and editing the manuscript. All authors read and approved the final manuscript.

## Ethics Statement

This study was reviewed and approved by Ethics Committee of Shantou University Medical College (Cancer Hospital of SUMC, Approval number: 2023101). We confirm that we have the necessary consents from all patients/participants involved in the study.

## Conflicts of Interest

The authors declare no conflicts of interest.

## Supporting information

Supporting information.

## Data Availability

The data presented in this study are available on request from the corresponding author.
